# Candida parapsilosis Fungemia in a Patient With Spinal Cord Injury on Sodium-Glucose Cotransporter-2 Inhibitor Therapy: A Case Report

**DOI:** 10.7759/cureus.88907

**Published:** 2025-07-28

**Authors:** Ashley E Traczuk, Michael Stillman

**Affiliations:** 1 Medicine, Thomas Jefferson University Hospital, Philadelphia, USA; 2 Internal Medicine, Thomas Jefferson University Hospital, Philadelphia, USA

**Keywords:** candida parapsilosis, case report, cervical spinal cord injury, fungemia, sodium-glucose transport-2 inhibitor, spinal cord injury

## Abstract

Individuals with spinal cord injury (SCI) and neurogenic bladder often rely on catheterization programs, which increase the risk of urinary tract infections (UTIs). Sodium-glucose cotransporter-2 inhibitors (SGLT2i), widely used in the management of diabetes mellitus (DM), chronic kidney disease (CKD), and congestive heart failure (CHF), promote urinary glucose excretion, potentially fostering an environment favorable to bacterial and fungal growth. This case describes a 53-year-old man with long-standing traumatic cervical SCI and a suprapubic catheter who presented with sepsis secondary to *Candida parapsilosis* fungemia, in the context of SGLT2i use for DM management. This report suggests that the combination of SGLT2i therapy and chronic catheterization may increase the risk of urinary tract and invasive fungal infections. While current evidence is lacking, further investigation is warranted, and clinicians should exercise caution when prescribing SGLT2i in patients with SCI and bladder catheter use.

## Introduction

Individuals with spinal cord injury (SCI) are at increased risk for cardiometabolic diseases, including atherosclerosis, dyslipidemia, and diabetes mellitus (DM) [1-3]. A large-scale study (n = 60,678) reported that individuals with SCI have nearly double the risk of developing DM compared to those without such injuries [4]. Other studies have similarly documented significantly higher rates of insulin resistance and type 2 DM among the SCI population [5-7]. Although the underlying mechanisms are not fully understood, injury-induced sarcopenia is thought to play a major role. Muscle loss contributes to insulin resistance, which in turn may promote atherogenesis, metabolic syndrome, and steatohepatitis [8].

Bladder management is another key concern for individuals with SCI. Nearly all patients require either an indwelling urinary catheter (IUC) or clean intermittent catheterization (CIC) for adequate bladder drainage. One study found that nearly 7 of 10 individuals with SCI relied on one of these two methods [9]. While there is ongoing debate about the optimal voiding strategy, often influenced by caregiver availability, hand function, body habitus, and patient preference [10], bladder catheterization is a well-established risk factor for urinary tract infections (UTIs), increasing the odds more than twofold on top of the elevated baseline risk associated with SCI itself [11].

Sodium-glucose cotransporter-2 inhibitors (SGLT2i) are increasingly prescribed for the management of type 2 DM, congestive heart failure (CHF), and chronic kidney disease (CKD) [12]. Although most large reviews have not found a significantly increased risk of UTI with SGLT2i use, these agents are clearly associated with external genital fungal infections [13-15], and isolated reports have described cases of invasive fungal infections, including fungemia [16,17]. In this report, we describe the case of a 53-year-old man with chronic cervical SCI, complicated by DM, hypertension (HTN), atherosclerotic heart disease, and neurogenic bladder managed with a suprapubic catheter. After starting treatment with an SGLT2i, he developed an upper urinary tract *Candida parapsilosis* infection leading to fungemia.

## Case presentation

Patient information

A 53-year-old man with a history of traumatic C4 spinal cord injury (American Spinal Injury Association (AIS) grade C), sustained 11 years prior, presented to the emergency department with confusion, lethargy, and hypotension. His medical history was notable for non-ST elevation myocardial infarction (MI), type 2 diabetes mellitus (DM) with proteinuria (maximum urine protein-to-creatinine ratio: 785 mg/g), hypertension (HTN), depression, neurogenic bowel, and neurogenic bladder. His diabetes had previously been managed with metformin alone; however, in response to rising glycohemoglobin (HbA1c) levels, peaking at 7.7%, and worsening proteinuria, he was recently initiated on empagliflozin, a sodium-glucose cotransporter-2 inhibitor (SGLT2i). Although he had been offered dulaglutide, a glucagon-like peptide-1 receptor agonist (GLP-1 RA), he declined its use based on concerns from his home care nurse about a potential risk of thyroid cancer. His neurogenic bladder was managed with a suprapubic catheter, routinely maintained by the hospital’s neuro-urology service. He experienced occasional mild bladder spasms, which were effectively controlled with oral oxybutynin administered three times daily.

Clinical findings

**Figure 1:**
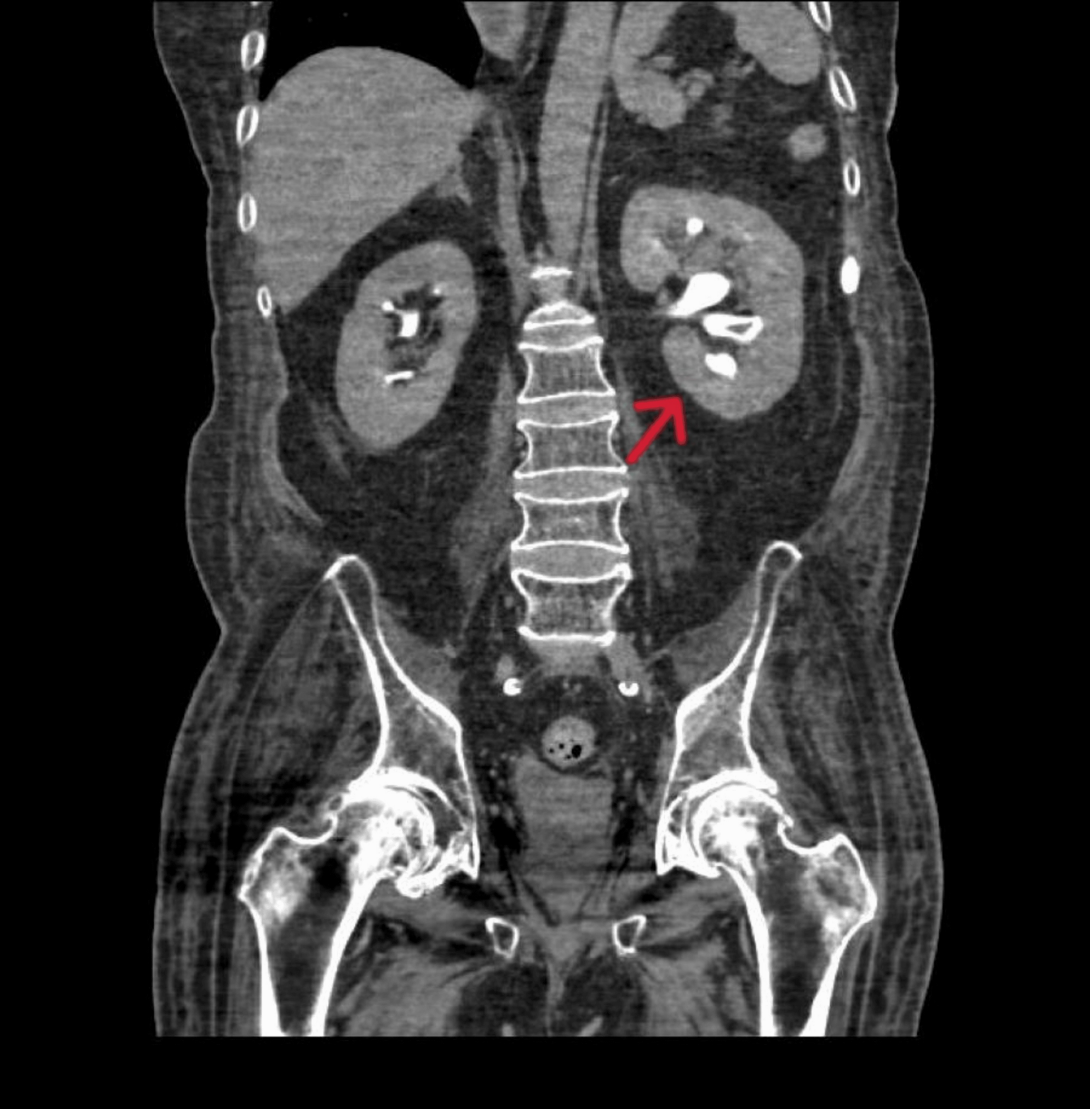
CT urogram demonstrating mild left-sided hydroureteronephrosis with urothelial thickening

**Figure 2:**
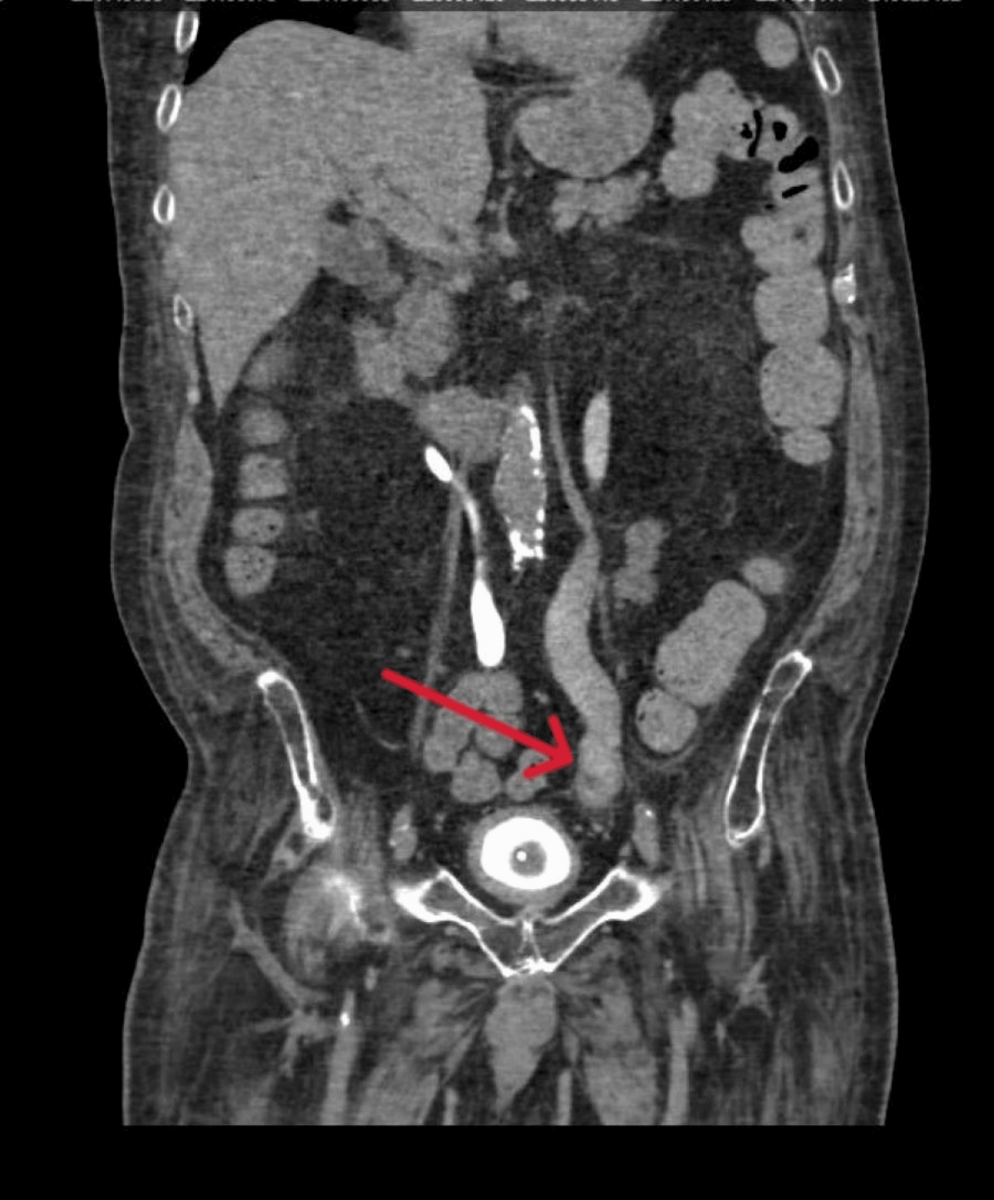
Left distal ureter enhancement with periureteric fat stranding with intraluminal filling defect

In the emergency department, the patient was febrile at 39.4℃, tachycardic with a heart rate of 124 bpm, and normotensive with a blood pressure of 116/68 mmHg. On physical examination, he was disoriented but had no abdominal tenderness. Urinalysis revealed 4+ glucose (likely secondary to SGLT2i use), 2+ ketones, and 26 white blood cells per high-power field. He was admitted for further evaluation and management, and several home medications, including empagliflozin, were withheld. As part of the diagnostic workup, contrast-enhanced CT of the abdomen and pelvis revealed mild left-sided hydroureteronephrosis, urothelial thickening and hyperenhancement, and bladder wall thickening, without evidence of an obstructing lesion. Given concerns for urosepsis, blood cultures were obtained, and empirical intravenous fluids and broad-spectrum antibiotics were initiated. A urology consultation was also requested. Subsequent CT urography showed enhancement of the distal left ureter with adjacent periureteric fat stranding and an intraluminal filling defect concerning for neoplasm. Cystoscopy was performed and revealed a normal-appearing bladder; however, a filling defect was visualized in the distal left ureter, raising suspicion for a fungal ball (Figure 1 and Figure 2). Specimens retrieved during the procedure were sent for histopathological analysis, which demonstrated fungal elements and debris. Both blood and urine cultures grew *Candida parapsilosis*, a fungal organism known to thrive in glucose-rich environments and frequently associated with colonization of intravenous lines and indwelling catheters [18,19].

Therapeutic intervention/follow-up

The patient was treated with an 8-week course of fluconazole, and empagliflozin was permanently discontinued. At the 8-week mark, he was evaluated by an infectious diseases specialist, who recommended extending antifungal therapy for an additional two weeks. A surveillance urine culture obtained at that time showed several non-dominant bacterial organisms consistent with bladder colonization, but no fungal elements. Consequently, fluconazole therapy was discontinued.

## Discussion

In this article, we present the case of a 53-year-old man with SCI, type 2 diabetes mellitus, coronary artery disease, and neurogenic bladder who developed invasive fungal disease and severe sepsis after initiating treatment with an SGLT2i. Several aspects of this case merit discussion.

The patient was prescribed an SGLT2i only after declining a GLP-1 receptor agonist, a decision influenced by misinformation. Given his comorbidities, including cardiovascular disease and a BMI of 27.6, either agent would have been appropriate and potentially beneficial [12,20]. However, comprehensive counseling regarding the risks and benefits of each option may have supported a more informed decision. Clarification of the misinformation he received, ideally from a trusted provider such as his primary care physician, might have led him to choose a GLP-1 agonist and potentially avoid complications associated with SGLT2i use.

Although several case reports have described invasive fungal infections associated with SGLT2i [13-17], data on the true incidence of this complication remain lacking. Clinicians should remain vigilant about this underrecognized risk, especially in patients with SCI, who already face a high burden of urinary tract infections, with an annual incidence of 2.72 episodes per 100 patient-days [11].

## Conclusions

There is a notable lack of data assessing the efficacy, risks, and benefits of SGLT2i use in individuals with SCI. Given the high prevalence of cardiometabolic disease in this population, many patients may be appropriate candidates for and benefit from SGLT2i therapy. However, their risk of adverse effects, particularly infectious complications, may exceed that of the general population. While a meta-analysis of SGLT2i-related infections found no significant increase in UTIs, the included study populations did not consist of individuals at elevated risk for bladder or upper tract infections. As such, these findings may not be generalizable to patients with SCI and neurogenic bladder, most of whom rely on intermittent or indwelling catheterization. Until the specific risks of SGLT2i use in this group are better understood, clinicians should approach prescribing these agents with caution. Focused research is needed to clarify the safety profile and optimize the therapeutic use of SGLT2 inhibitors in this vulnerable population.
